# Eculizumab use in a tertiary care nephrology center: data from the Vienna TMA cohort

**DOI:** 10.1007/s40620-021-00981-8

**Published:** 2021-02-18

**Authors:** Christof Aigner, Martina Gaggl, Gunar Stemer, Michael Eder, Georg Böhmig, Renate Kain, Zoltán Prohászka, Nóra Garam, Dorottya Csuka, Raute Sunder-Plassmann, Leah Charlotte Piggott, Natalja Haninger-Vacariu, Alice Schmidt, Gere Sunder-Plassmann

**Affiliations:** 1 Division of Nephrology and Dialysis, Department of Medicine III, Medical University Vienna, Vienna, Austria; 2 Department of Pharmacy, Vienna General Hospital, Vienna, Austria; 3 Department of Pathology, Medical University Vienna, Vienna, Austria; 4 Research Group for Immunology and Haematology, Department of Internal Medicine and Hematology, Semmelweis University- Eötvös Loránd Research Network (Office for Supported Research Groups), Budapest, Hungary; 5 Genetics Laboratory, Department of Laboratory Medicine, Medical University Vienna, Vienna, Austria

**Keywords:** Genetic renal disease, Hemolytic uremic syndrome, Thrombotic microangiopathy, Eculizumab

## Abstract

**Background:**

Practice patterns of eculizumab use are not well described. We examined indications for, and outcomes of, eculizumab therapy in a tertiary care nephrology center.

**Methods:**

We used the “Vienna TMA cohort” and the hospital pharmacy database at the Medical University of Vienna to identify patients that received eculizumab treatment between 2012 and 2019. We describe clinical characteristics, details of eculizumab use, and outcomes of patients with complement gene-variant mediated TMA (cTMA), secondary TMA (sTMA) and C3 glomerulopathy (C3G).

**Results:**

As of December 2019, 23 patients received complement blockade at the Division of Nephrology and Dialysis: 15 patients were diagnosed with cTMA, 6 patients with sTMA and 2 patients with C3G. Causes of sTMA were bone marrow transplantation (*n* = 2), malignant hypertension, malignant tumor, systemic lupus erythematosus, antiphospholipid syndrome and lung transplantation (each *n* = 1). Across all indications, patients had a median age of 31 and were predominantly female (78%) and the median duration of treatment was 227 days. Hematological recovery was seen in most patients, while renal response was best in patients with cTMA. Adverse events were recorded in 26%.

**Conclusions:**

In summary, eculizumab is the treatment of choice for cTMA patients that do not respond to plasma therapy. In patients with sTMA and C3G, the response rates to therapy are much lower and therefore, the decision to start therapy needs to be considered carefully.

**Graphic abstract:**

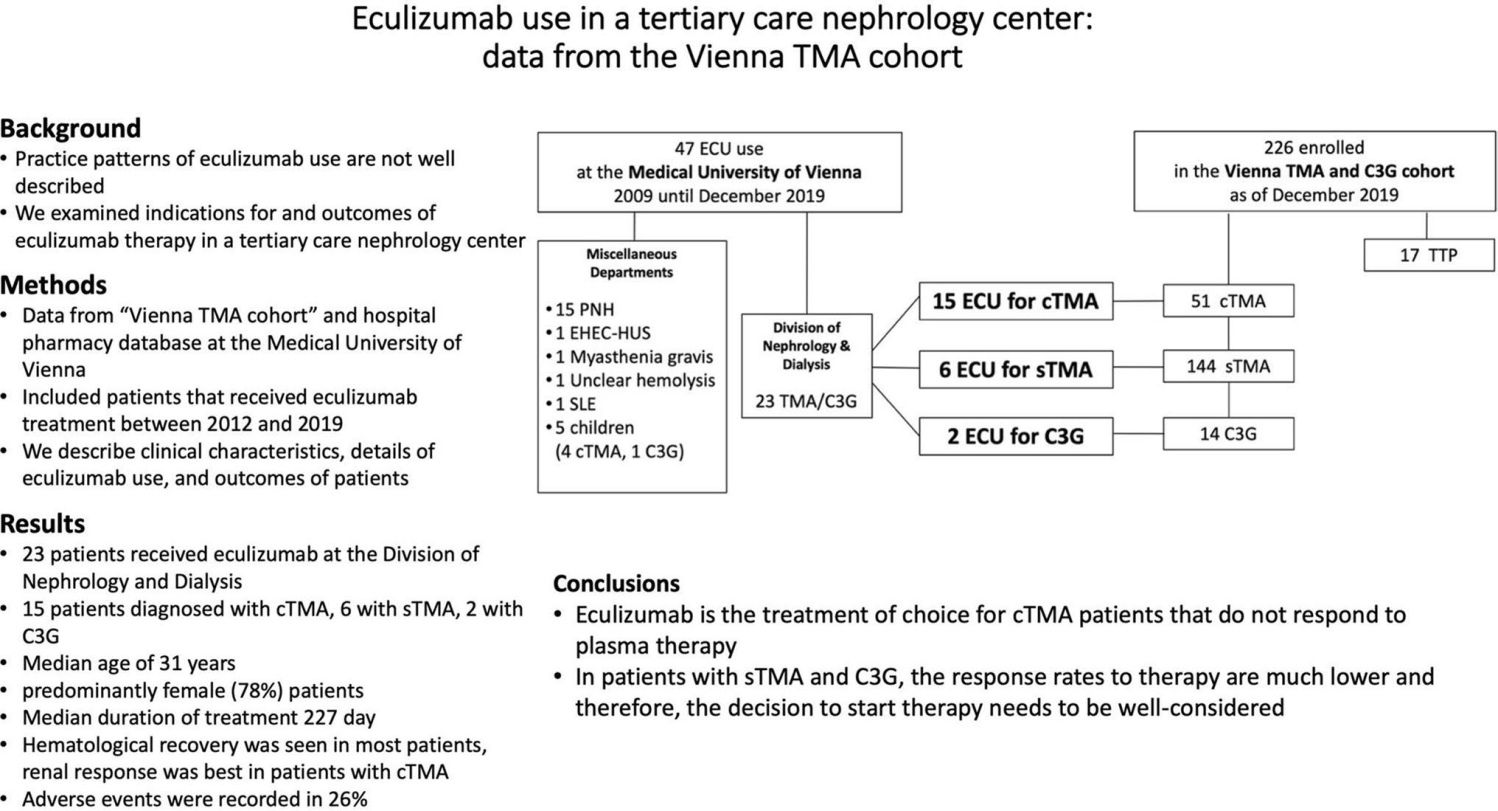

**Supplementary Information:**

The online version contains supplementary material available at 10.1007/s40620-021-00981-8.

## Introduction

Eculizumab is a recombinant humanized monoclonal IgG2/4 antibody that binds to the human C5 complement protein and inhibits the activation of the terminal complement complex. It is approved for treatment of paroxysmal nocturnal hemoglobinuria (PNH), myasthenia gravis (MG), and atypical hemolytic uremic syndrome (aHUS)/complement gene-variant mediated thrombotic microangiopathy (cTMA) [1].

Defective complement regulation causes the kidney diseases cTMA and C3-glomerulopathy (C3G). These diseases are characterized by endothelial and mesangial cell damage due to dysregulation of the alternative complement cascade on cell surfaces in cTMA, and in C3G by the deposition of complement components in the mesangium and subendothelial space, or within the glomerular basement membrane [2]. Secondary TMAs (sTMAs) are caused by other underlying conditions, such as infections, autoimmune disease, or medical treatment. Ultimately, cessation of those triggers should lead to resolution of the TMA [3].

Thrombotic microangiopathies and C3G frequently lead to end-stage renal disease (ESRD) or death if untreated. Eculizumab use has tremendously improved kidney and patient survival in patients with cTMA. In contrast to cTMA, eculizumab is not licensed for use in patients with C3G or sTMA. However, a few case reports or case series have shown favorable outcomes in patients with C3G or sTMA [4, 5].

Because therapy with eculizumab bears a certain risk for life threatening infectious diseases, is costly for National Health Systems and cumbersome for patients, some experts recommend cessation of treatment in patients with cTMA depending on the risk of disease recurrence [6, 7]. In this context, practice patterns of Eculizumab use in patients with TMA and C3G are not well described. We therefore used the Vienna TMA cohort and the hospital pharmacy database at the Medical University of Vienna to identify patients with a history of Eculizumab use between 2012 and 2019 [8–12]. We describe the clinical characteristics, dose and duration of eculizumab therapy, and the outcomes of patients with cTMA, sTMA, and C3G.

## Methods

### Design and study population

The Vienna TMA cohort was established in 2014 at the Division of Nephrology and Dialysis, Department of Medicine III, Medical University of Vienna [8, 9]. We include patients with a diagnosis of any form of TMA and other complement-mediated kidney diseases. In this study we describe the clinical characteristics and outcomes of all consecutive patients from our department who received eculizumab for the treatment of cTMA, sTMA, and C3G since 2012.

### Data source

We identified all patients who received eculizumab at the Medical University of Vienna using the electronic records of the Department of Pharmacy and matched these data with the Vienna TMA cohort. Demographic, clinical, and genetic data were retrieved from the electronic and paper-based health care records of our institution. For patients that were lost to follow-up, no information on laboratory data was available. However, information concerning patient death and renal replacement therapy was available through the Austrian Dialysis and Transplant Registry (OEDTR). The institutional review board at the Medical University of Vienna approved the study (identifier: 1368/2014). Patients that were prospectively included in the study provided written consent.

### Definitions

Thrombotic microangiopathy was diagnosed in the presence of mechanical hemolytic anemia, thrombocytopenia and acute or worsening kidney injury. Hematologic response was defined as an increase in both hemoglobin and platelet count during therapy with eculizumab. Furthermore, improvement of chronic kidney disease (CKD) stage was also examined. Further information on definitions, cut-off values for laboratory results and CKD stages can be found in the supplemental information.

### Laboratory methods

The standard laboratory work-up was performed at the Department of Laboratory Medicine, Medical University of Vienna. Genetic analysis was performed at the Department of Laboratory Medicine at the Medical University of Vienna and the research laboratory of the Department of Internal Medicine, Semmelweis University [9, 13].

### Statistical analysis

Data are presented as count and frequency, as mean and standard deviation or as median and interquartile range, as appropriate. We used MS Excel and IBM SPSS 24.0 for data management and analysis and GraphPad Prism 8.4. for the generation of figures.

## Results

### Patients

**Fig. 1:**
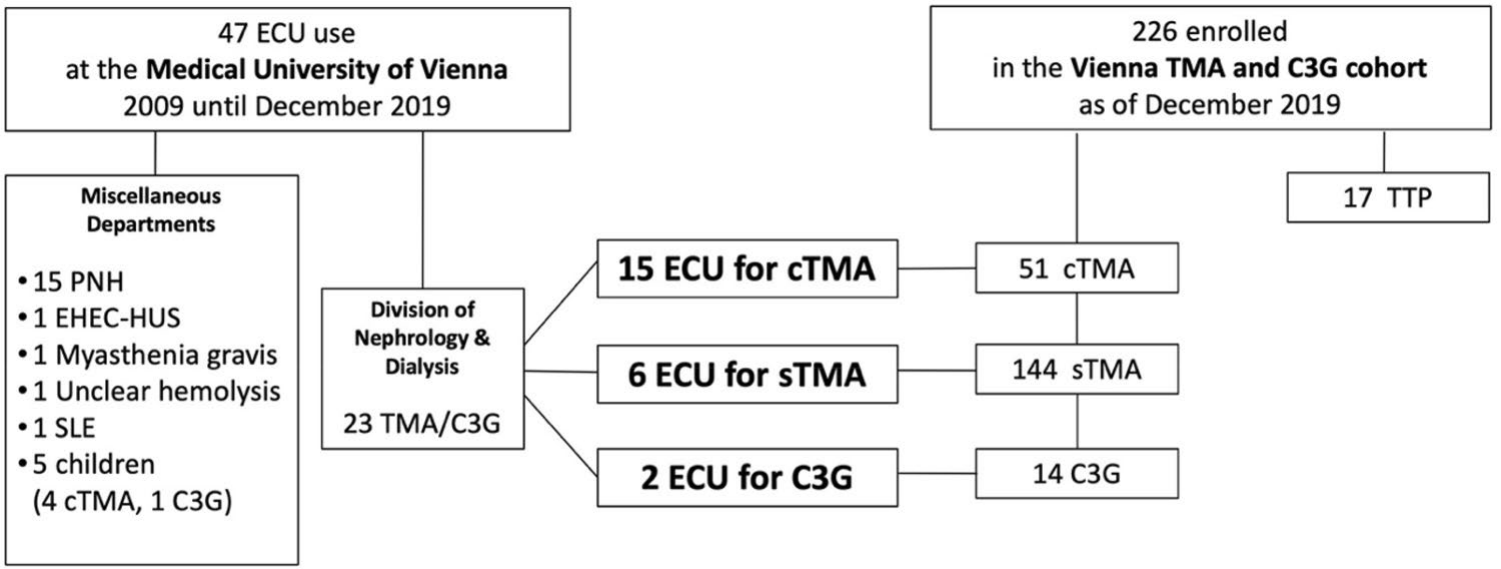
Patients treated with eculizumab at the Medical University of Vienna. ECU, eculizumab; TMA, thrombotic microangiopathy; C3G, C3 glomerulopathy; PNH, paroxysmal nocturnal hemoglobinuria; EHEC-HUS, enterohemorrhagic Escherichia coli—hemolytic uremic syndrome; SLE, systemic lupus erythematosus; cTMA, complement gene-variant mediated thrombotic microangiopathy; sTMA, secondary thrombotic microangiopathy; TTP, thrombotic thrombocytopenic purpura

We identified a total of 47 patients that received eculizumab at the Medical University of Vienna from 2009 until 2019 (Fig. 1, supplemental information).

Between 2012 and 2019, a total of 23 patients (6 retrospectively between 2012 and 2014, and 17 prospectively after 2014) consecutively received eculizumab at our Department for the treatment of cTMA (*n* = 15, 65%), sTMA (*n* = 6, 26%) and C3G (*n* = 2, 9%). The majority of patients were female (78%) and median age at eculizumab initiation was 31 years. Two patients had a family history for complement-mediated diseases. Triggering factors for secondary TMA were bone marrow transplantation in two cases and malignant hypertension, solid organ transplantation, carcinoma and catastrophic antiphospholipid syndrome (CAPS) in one patient each. Of the two patients diagnosed with C3G, one was diagnosed after kidney transplantation (KTX). Investigation of complement-gene variants were performed in all patients (Table 1). Anti-CFH autoantibodies were measured in all patients and no positive results were reported in eculizumab treated patients. Eculizumab was administered as per the manufacturer’s instructions (900 mg weekly for 4 weeks, followed by 1200 mg every second week starting on week 5) [1]. Across all indications, the median duration of eculizumab therapy was 227 days (interquartile range (IQR): 68–966 days). One patient diagnosed with C3G in the renal graft died shortly after initiation of therapy and therefore only received 2 doses of eculizumab. The baseline characteristics of all patients are detailed in Table 1. Twenty patients underwent a kidney biopsy at initial disease presentation. Median time of follow-up was 1,875 (IQR: 1,003–3,380) days from the first disease episode and 1,186 (876–1,939) days from the first eculizumab dose, respectively. In total, 2 patients (1 cTMA, 1 sTMA) were lost to follow-up and 4 patients died during follow-up.

**Table 1:** Patient characteristics

Characteristic	All patients	cTMA	sTMA	C3G
No. of patients	23	15	6	2
Female	18 (78.3%)	12 (80%)	5 (71.4%)	2 (100%)
Race				
Caucasian	21 (91.3%)	14 (93%)	6 (100%)	1 (50%)
Asian	2 (8.7%)	1 (6.6%)	0	1 (50%)
Family history of TMA/C3G	2 (8.7%)	2 (13%)	n.a	0
Age at initial disease presentation (years)	28 (20–46)	27 (19–46)	30 (25–43)	26/51^a^
Age at Ecu initiation (years)	31 (25–46)	31 (24–46)	30.5 (25.5–42)	29/54^a^
History of KTX	6 (26%)	5 (33.3%)	0	1 (50%)
Time to Ecu from first diagnosis (days)	42 (15–1,662)	813 (14–4,166)	22 (16–29)	994/1,117
Time to Ecu from start of current flare (days)	19 (7–28)	15 (6–20)	22 (16–29)	38/994
PE/PI before Ecu initiation	18 (78.3%)	13 (86.7%)	5 (83.3%)	0
Duration of Ecu therapy (days)	227 (68–966)	490 (151–1,745)	99 (24–229)	14/246^a^
Deceased during FU	4 (17.4%)	2 (13.3%)	1 (16.6%)	1 (50%)
Genetic complement variant^a^				
* CFH*	3 (13%)	3 (20%)	0	0
* CFI*	1 (4.3%)	1 (6.6%)	0	0
* C3*	3 (13%)	3 (20%)	0	0
* CD46*	1 (4.3%)	1 (6.6%)	0	0
* CFHR5*	1 (4.3%)	1 (6.6%)	0	0
Combined variants	2 (8.7%)	2 (13.3%)	0	0
*CFH*-H3				
Homozygous	6 (26%)	4 (26.6%)	2 (33.3%)	0
Heterozygous	10 (43.5%)	7 (46.6%)	1 (16.6%)	2 (100%)
*CD46*ggaac				
Homozygous	7 (30.4%)	6 (40%)	1 (16.6%)	0
Heterozygous	11 (48%)	6 (40%)	4 (66.6%)	1 (50%)

Numbers are count and percent or median and interquartile range

*cTMA* complement gene-variant mediated thrombotic microangiopathy, *sTMA* secondary thrombotic microangiopathy, *C3G* C3-glomerulopathy, *Ecu* eculizumab, *FU* follow-up, *PE* plasma exchange, *PI* plasma infusion

^a^Only pathogenic or likely pathogenic variants were included

### Eculizumab use in patients presenting with cTMA

Among the 15 patients with cTMA who were treated with eculizumab, 80% were female and five (33.3%) had previously received a renal transplant. Time from first diagnosis of cTMA to initiation of eculizumab therapy varied greatly (2–8439 days). Seven patients received eculizumab during their first disease flare and eight had already been diagnosed with cTMA in the past. Thirteen (86.6%) patients received plasma exchange (PE) or plasma infusions (PI) before initiation of eculizumab. Of these 13, 6 had been diagnosed with cTMA in the past and were switched to eculizumab because of non-response to plasma therapy in 3 cases and allergic reactions to plasma infusions in the other 3. Two patients received eculizumab without previous plasma therapy because of known resistance to plasma therapy and patient preference, respectively. The median duration of eculizumab therapy was 490 days and five (33.3%) patients were still on therapy at the last follow-up. Of note, the kidney transplant recipients received eculizumab treatment from 12 days to 6 years after TX, due to either relapse of cTMA in 4 cases or to intolerance to prophylactic plasma therapy in 1 case. To date, none of the patients that experienced cTMA relapse after KTX and were treated with eculizumab lost the graft. Fourteen (93%) patients underwent kidney biopsy at initial disease presentation and in 3 patients, a kidney biopsy was performed before starting eculizumab therapy.

### Hematologic response

**Fig. 2:**
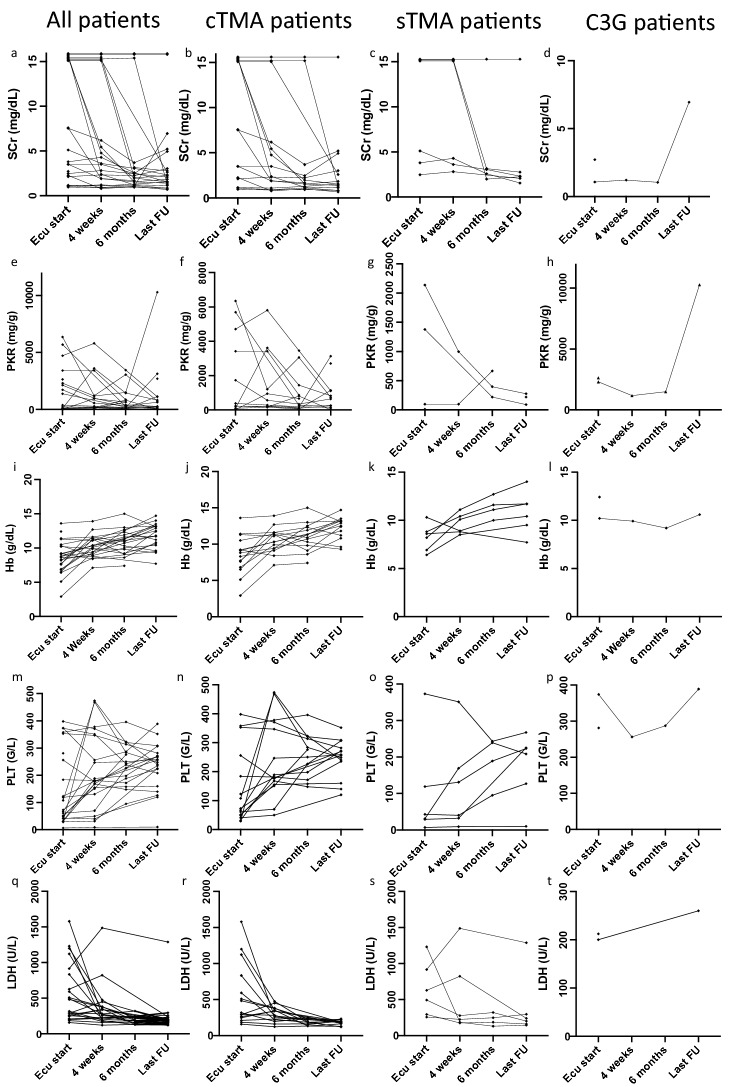
Hematologic and renal follow-up during therapy with eculizumab. The lines all contain different laboratory values of kidney function and parameters of hemolysis. The different patient cohorts are shown separately in each column. cTMA, complement gene-variant mediated thrombotic microangiopathy; sTMA, secondary thrombotic microangiopathy; C3G, C3 glomerulopathy; SCr, serum creatinine; PKR, urinary protein to creatinine ratio; Hb, hemoglobin; PLT, platelet count; LDH, lactate dehydrogenase; Ecu, eculizumab; FU, follow-up. A serum creatinine of 15 mg/dL means that patients were dependent on renal replacement therapy

At baseline, more than 90% of patients had anemia with a mean hemoglobin concentration of 8.5 g/dL. In contrast, thrombocytopenia was less common. After 4 weeks of eculizumab therapy, both the mean hemoglobin concentration and platelet counts showed a marked increase and remained stable until the last follow-up visit in all patients (Table 2; Fig. 2). Correspondingly, lactate dehydrogenase (LDH) levels markedly decreased after 4 weeks of therapy and remained low during the whole observation time.

**Table 2:** Hematological and renal response to eculizumab therapy in 15 cTMA patients

Characteristic	Baseline	4 weeks	6 months	Last FU
Anemia	14 (93.3%)	13 (86.7%)	11 (84.6%)	7 (46.6%)
Mean Hb (g/dL)	8.5 (± 2.7)	10.5 (± 1.7)	11 (± 2)	12.2 (± 1.5)
Thrombopenia	10 (66.7%)	2 (13.3%)	1 (7.7%)	1 (8.3%)
Mean PLT count (G/L)	145 (± 131)	240 (± 135)	253 (± 74)	246 (± 65)
Mean LDH (U/L)	552 (± 436)	301 (± 105)	192 (± 41)	181 (± 33)
AKI	12 (80%)	n.a	n.a	n.a
RRT	6 (40%)	3 (20%)	2 (14.3%)	1 (7.7%)
Mean SCr (mg/dL)	3.3 (± 2.6)	2.67 (± 1.88)	1.73 (± 0.79)	2.06 (± 1.49)
Mean PKR (mg/g)	1,872 (± 2,391)	1,505 (± 1,907)	947 (± 1,155)	851 (± 998)
KTX	4 (26.7%)	4 (26.7%)	4 (26.7%)	5 (33.3%)
Patients on Ecu therapy	15 (100%)	15 (100%)	11 (67%)	5 (33.3%)
Lost to FU	0	2 (13.3%)	2 (13.3%)	3 (20%)

Numbers are count and percent or mean and standard deviation. No 4 week + 6mo FU data for 2 (only lab results). No last FU lab data for 3 pat. Anemia was defined as Hb levels of < 12.5 g/dL for female and < 13.5 g/dL for male patients. Thrombopenia was defined as platelet count < 150 G/L

*cTMA *complement gene-variant mediated thrombotic microangiopathy, *FU* follow-up, *Hb* hemoglobin, *PLT* platelets, *LDH* lactate dehydrogenase, *SCr* serum creatinine, *PKR* protein-creatinine ratio, *RRT* renal replacement therapy, *KTX* kidney transplantation, *Ecu* eculizumab

### Renal response

**Fig. 3:**
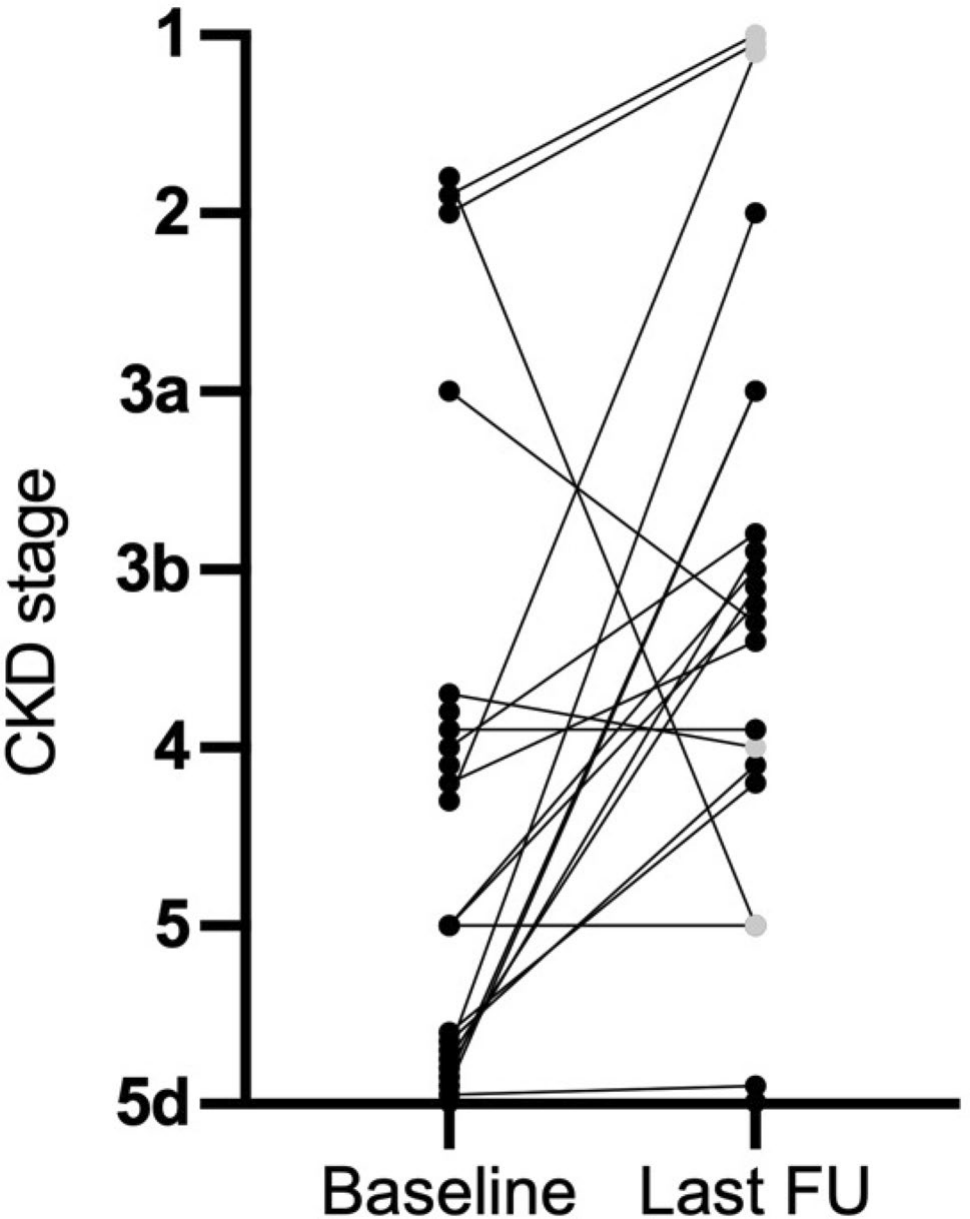
Development of CKD stages in patients treated with eculizumab. Silver dots indicate that patients were still receiving eculizumab at the last follow-up. CKD, chronic kidney disease; FU, follow-up

At eculizumab initiation, 12 (80%) patients suffered from acute kidney injury (AKI) and six (40%) received renal replacement therapy (RRT). After 4 weeks of therapy, RRT could be discontinued in three patients, while three others remained on chronic renal replacement therapy. For the patients not on renal replacement therapy, mean serum creatinine at baseline was 3.3 mg/dL, which continued to decline after 4 weeks and 6 months of therapy. The same trend was seen for the urine protein to creatinine ratio. In total, the CKD stage of eight patients (53.3%) had improved by at least one level after 6 months (Tables 2, 3, 4; Fig. 3). The remaining patients (that were not dependent on RRT) had stable CKD stages and none of the patients showed a significant loss of eGFR.

**Table 3:** Hematological and renal response to eculizumab therapy in six sTMA patients

Characteristic	Baseline	4 weeks	6 months	Last FU
Anemia	6 (100%)	6 (100%)	4 (66.6%)	5 (83.3%)
Mean Hb (g/dL)	8.2 (± 1.4)	9.6 (± 1)	11.4 (± 1.1)	10.8 (± 2.2)
Thrombopenia	5 (83.3%)	4 (66.7%)	1 (25%)	2 (33.3%)
Mean PLT count (G/L)	100 (± 139)	122 (± 128)	191 (± 69)	177 (± 94)
Mean LDH (U/L)	635 (± 378)	528 (± 530)	221 (± 82)	388 (± 445)
AKI	6 (100%)	n.a	n.a	n.a
RRT	4 (66.7%)	3 (50%)	1 (16.6%)	1 (16.6%)
Mean SCr (mg/dL)	3.8 (± 1.87)	3.56(± 0.74)	2.69 (± 0.54)	2.18 (± 0.44)
Mean PKR (mg/g)	694 (± 969)	n.a.^a^	428 (± 226)	196 (± 95)
KTX	0	0	0	0
Patients on Ecu therapy	6 (100%)	4 (66.6%)	2 (33.3)	0
Lost to FU	0	0	0	2 (33.3%)

Numbers are count and percent or mean and standard deviation

*sTM* secondary thrombotic microangiopathy, *FU* follow-up, *Hb* hemoglobin, *PLT* platelets, *LDH* lactate dehydrogenase, *SCr* serum creatinine, *PKR* protein-creatinine ratio, *RRT* renal replacement therapy, *KTX* kidney transplantation, *Ecu* eculizumab

^a^Data only available for 1 patient. Anemia was defined as Hb levels of < 12.5 g/dL for female and < 13.5 g/dL for male patients. Thrombopenia was defined as platelet count < 150 G/L

**Table 4:** Cessation of eculizumab therapy

Characteristic	4 weeks	6 months	Last FU
cTMA			
Therapy terminated	0	4 (26.7%)	10 (66.6%)
Weaning after response	0	1 (7.7%)	4 (26.6%)
Non-response	0	1 (7.7%)	1 (7.7%)
Patient preference	0	2 (13.3%)	0
Patient death^a^	0	0	1(7.7%)
sTMA			
Therapy terminated	2 (33.3%)	2 (33.3%)	6 (100%)
Weaning after response			2 (33.3%)
Non-response	2 (33.3%)	2 (33.3%)	n.a
C3G			
Therapy terminated	1 (50%)	0	2 (100%)
Patient death^a^	1 (50%9)	0	0
Non-response	0	0	1 (50%)

*FU* follow-up, *cTMA* complement gene-variant mediated thrombotic microangiopathy, *sTMA* secondary thrombotic microangiopathy, *C3G* C3-glomerulopathy

^a^Causes of death: 1 unknown, 1 pulmonary embolism

### Eculizumab use in patients presenting with sTMA

Six patients with a diagnosis of secondary TMA received eculizumab in our center. The median time from first diagnosis to the initiation of eculizumab therapy was 22 days (IQR 16–29). All patients had experienced a first episode of TMA and five (83.3%) had received PE or PI before the initiation of terminal complement blockade (Table 2). One patient received eculizumab without prior plasma therapy since hemolysis had ceased spontaneously 3 days before the diagnosis of TMA by kidney biopsy. Four (66.6%) patients underwent kidney biopsy and all of them showed features of chronic TMA. Neither of the patients presenting after bone marrow transplantation received an immunosuppressant drug at the time of TMA diagnosis.

### Hematologic response

All patients were anemic at baseline and five of the six patients were thrombocytopenic, with both lower mean hemoglobin and platelet counts as compared to cTMA patients at initiation of therapy with eculizumab. Improvement in hematologic parameters was less pronounced in sTMA patients, but a rise in both hemoglobin concentration and platelet counts was seen 4 weeks after initiation of therapy. No improvement in platelet counts after the initiation of therapy was seen in the two patients with sTMA following bone marrow transplantation (Table 3).

### Renal response

At baseline, all patients suffered from AKI and four (66.7%) were on renal replacement therapy. After 4 weeks of therapy, RRT could be discontinued in one patient and in another after 6 months of therapy. One patient recovered renal function after 6 months, but received eculizumab therapy for only approximately 3 months. The mean serum creatinine for the two patients not on renal replacement therapy was 3.8 mg/dL at baseline and decreased slightly after 4 weeks and 6 months of follow-up (Table 3). After 6 months, four (60%) patients improved by at least one CKD stage (Supplemental Table 2).

### Eculizumab use in patients presenting with C3G

Two patients diagnosed with C3G were treated with eculizumab (Table 2).

One patient, who had TMA as a primary kidney disease and was diagnosed with C3G in the kidney graft, died after 2 weeks of therapy from massive pulmonary embolism. Therefore, no information on treatment response is available (Fig. 2). The serologic complement profile of this patient showed a slight elevation of soluble C5b-9.

The other patient received eculizumab for a total of 8 months because C3G was refractory to other treatments. Previous therapies included treatment with ACE-inhibitors, intravenous immunoglobulins, rituximab, corticosteroids and mycophenolic acid. Serum creatinine was 1.07 mg/dL at initiation of eculizumab and remained stable during the entire treatment period. Urinary protein to creatinine ratio was 2308 mg/g at the start of therapy and remained at that level for the entire duration. A renal biopsy was performed to assess treatment response on the day of the last eculizumab infusion. This confirmed the diagnosis of C3G, but showed more chronic damage (glomerular scars, interstitial fibrosis) as compared to a previous biopsy three years earlier (Fig. 2; Table 4). This patient showed low levels of C3, deficient alternative pathway activity and high levels of soluble C5b-9. However, C3 nephritic factor was negative.

### Weaning off eculizumab

Of the 15 cTMA patients who commenced eculizumab, 11 were still on treatment after 6 months and five were on long-term therapy until last follow-up (Table 4). None of the patients experienced a cTMA relapse after cessation of eculizumab therapy.

Reasons for cessation of therapy were weaning after response in one patient, non-response in another and patient preference in two other cases. One patient died under therapy at home due to unknown causes.

None of the sTMA patients were on long-term eculizumab therapy. In four of these patients, eculizumab was stopped due to non-response after 4 weeks and 6 months of therapy. Two responded to treatment and eculizumab was stopped after 6 months of treatment. Both of these patients had stable renal function until the last follow-up.

### Adverse events

Adverse events were recorded in 26% of patients (Supplemental Table 2). One patient complained of worsening arterial hypertension, which led to cessation of therapy (patient preference). Blood pressure levels did not improve and he later died due to a ruptured aortic aneurysm. One acute liver injury occurred, during which eculizumab therapy was halted for several weeks and the dosing interval was subsequently extended to 1200 mg every 3 weeks. Further adverse events included transient exanthema and leukopenia, which both resolved spontaneously and did not lead to any alteration in drug dosing schedule. Two patients died under therapy, one due to pulmonary embolism shortly after drug initiation,, and the other during long-term treatment due to an unknown cause.

## Discussion

This analysis of patients with different diagnoses receiving eculizumab provides several insights that might help physicians with the decision to initiate eculizumab therapy in the future.

Eculizumab is a drug that is approved for the treatment of cTMA, and is used off-label for severe cases of sTMA, C3G and other indications. The high cost of the drug remains a challenge for public health systems and therefore, the indications to use eculizumab need to be well defined. Current KDIGO guidelines recommend starting eculizumab in all cases of cTMA. However, many experienced centers still start with plasma therapy when cTMA is suspected and only initiate eculizumab when plasma therapy fails [14]. In our study, 85% of patients with cTMA and 80% of patients with sTMA still received plasma therapy before eculizumab was started. This approach may reduce financial and patient burdens. Furthermore, KDIGO guidelines suggest lifelong treatment with eculizumab in patients with cTMA. In our cohort, eculizumab was continued in patients at high risk of relapse after discontinuation (*e.g. CFH* or *C3* variants) or with CKD stages IV and V (not on dialysis), because a relapse might lead to ESRD in those patients. However, administration of the drug was halted in individuals who responded well to treatment or were still dependent on RRT after approximately 6 months of treatment. In our cohort, 50% of cTMA patients showed markedly improved renal function after 6 months. Renal replacement therapy could be stopped in three patients after 4 months of treatment and in another patient after 6 months of treatment. Notably, patients with features of chronic TMA on the kidney biopsy and high serum creatinine at initial presentation showed less improvement in kidney function. Time from the beginning of the cTMA disease flare to initiation of complement blockade was a median of 15 days (IQR 6–20), which is in line with other studies [15]. Genetic variants were found in most patients diagnosed with cTMA, but in none of the patients with sTMA or C3G. Notably, *CFH* and *CD46* risk haplotypes were found in all patients, regardless of diagnosis. The significance of these findings has to be explored in future studies.

A total of six patients diagnosed with sTMA were treated with eculizumab. Median age at presentation was 30 years (IQR 19–46), which is younger than the average sTMA patient described in the literature [16]. All of these patients had severe TMA manifestations with lower mean hemoglobin levels and platelet counts as compared to the cTMA patients. Furthermore, all suffered from AKI and 66% of them were dependent on RRT. Five of six patients received plasma therapy before the initiation of complement blockade. Hence, young age, severity of TMA, and unresponsiveness to conventional treatment led to the decision to initiate eculizumab therapy in these patients. Mean treatment duration was shorter than in cTMA patients and none of the patients received long term complement blockade. Hematological response was seen in four of six patients. However, the two non-responsive patients had just undergone bone marrow transplantation before the treatment which could explain the lack of response. Concerning renal response, one patient was able to stop RRT after 4 weeks and another two after 6 months, however, two of these three patients received eculizumab for less than 4 months. In the setting of sTMA, only two patients showed a clear response to eculizumab treatment and were weaned off treatment after approximately 6 months of therapy. The other four patients showed no clear response and hence therapy was terminated. Larger observational reports of eculizumab administration across different types of sTMA are scarce and, in general, management of secondary TMAs is not well established due to a lack of general guidelines. Currently, most literature discusses TMA after bone marrow transplantation and reports concerning the effectiveness of eculizumab in this setting are conflicting because hematological and renal response may be promising, but overall survival is still poor [17–20]. The two patients that presented after bone marrow transplantation had a very different disease course. One patient had TMA some years after transplantation and regained renal function 4 months after termination of therapy with eculizumab, while the other showed no response to complement blockade and died shortly after diagnosis. The other patients received eculizumab related to malignant hypertension, solid organ transplantation, carcinoma and CAPS. The patient who was diagnosed with TMA after lung transplantation did not respond at all to eculizumab and remained dependent on RRT. The patients with TMA due to malignant hypertension and CAPS both responded well to therapy and were weaned off RRT. However, significant CKD persisted in both of these patients. In case of CAPS, eculizumab seems to be a viable treatment option, since the complement system is involved in the pathogenesis of the disease and case reports demonstrate good response to complement blockade. However, this suggestion is based only on case reports and series [21].

Two patients diagnosed with C3G received eculizumab in our cohort. One patient died of pulmonary embolism 2 weeks after therapy initiation, so no statement can be made concerning therapy response. The other patient received complement blockade because of gradual deterioration of kidney function after all other standard forms of care were unsuccessful. Both serum creatinine and urinary protein to creatinine ratio remained stable at a high level after 4 weeks and 6 months of therapy. In addition, more chronic lesions and sclerosis were diagnosed on a kidney biopsy. Therefore, the medication was stopped after 8 months of therapy. The patient progressed to ESRD in the following years. In general, eculizumab remains a treatment option in C3G, however, reported response rates in the literature are below 50% [22, 23].

Overall, 26% of patients experienced adverse events during eculizumab therapy. Two patients died while under therapy, but it is unclear whether the drug contributed to their death. One patient experienced drug-induced liver injury, which led to discontinuation of the drug, however, after switching to an extended dosing interval, liver enzymes remained in the reference range. One patient decided to discontinue eculizumab therapy due to worsening of arterial hypertension; another 2 patients showed exanthema and leukopenia, which disappeared spontaneously without halting the drug or changing the dosing schedule. Usually, eculizumab is a well-tolerated drug, however, severe adverse events can occur, including fatal infections and several rare adverse events. Therefore, the prescription needs to be deliberate [24–26].

In summary, eculizumab is the treatment of choice for cTMA patients that do not respond to conventional therapies. In patients with sTMA and C3G, the response rates to therapy are much lower and therefore, the decision to start the therapy needs to be considered carefully.

Due to the high cost of eculizumab and significant patient burden, some centers still perform plasma therapy first and only administer the drug after the failure of plasma therapy. The optimal duration of treatment and dosing schedule need to be decided on a patient to patient basis and still remain subject to future research. Patients diagnosed with sTMA or C3G may benefit from therapy. However, currently, there are no established clear-cut indications for complement blockade, and the high cost of treatment, the low response rates and the potentially serious side effects need to be considered carefully.

## Supplementary Information

Below is the link to the electronic supplementary material.

Supplementary file1 (DOCX 24 KB)

## Data Availability

The data that support the findings of this study are available from the corresponding author upon reasonable request.

## Abbreviations

aHUS: Atypical hemolytic uremic syndrome
OEDTR: Austrian Dialysis and Transplant Registry
C3G: C3-glomerulopathy
CKD: Chronic kidney disease
CKD-EPI: Chronic Kidney Disease Epidemiology Collaboration
cTMA: Complement gene variant mediated thrombotic microangiopathy
ESRD: End-stage renal disease
KDIGO: Kidney disease improving global outcomes
KTX: Kidney transplantation
MG: Myasthenia gravis
PNH: Paroxysmal nocturnal hemoglobinuria
RRT: Renal replacement therapy
