# Supplementary material for: Eculizumab use in a tertiary care nephrology center: data from the Vienna TMA cohort
Source: J Nephrol. 2021 Feb 18;35(2):451–61. doi: 10.1007/s40620-021-00981-8 (PMC8927043; doi:10.1007/s40620-021-00981-8)
Supplement: Supplementary file 1 — Supplementary file1 (DOCX 24 KB) [file 40620_2021_981_MOESM1_ESM.docx]

**Eculizumab use in a tertiary care nephrology center:**

**data from the Vienna TMA cohort**

**Supplementary Information**

Christof Aigner^1^, Martina Gaggl^1^, Gunar Stemer^2^, Michael Eder^1^, Georg Böhmig^1^,

Renate Kain^3^, Zoltán Prohászka^4^, Nora Garam^4^, Dorottya Csuka^4^, Raute Sunder-Plassmann^5^, Leah Charlotte Piggott^1^, Natalja Haninger-Vacariu^1^, Alice Schmidt^1^, Gere Sunder-Plassmann^1^

^1^Division of Nephrology and Dialysis, Department of Medicine III, Medical University Vienna, Vienna, Austria,

^2^Department of Pharmacy, Vienna General Hospital, Vienna, Austria,

^3^Department of Pathology, Medical University Vienna, Vienna, Austria,

^4^Research Laboratory, Department of Internal Medicine, and MTA-SE Research Group of Immunology and Hematology, Hungarian Academy of Sciences and Semmelweis University, Budapest, Hungary,

^5^Genetics Laboratory, Department of Laboratory Medicine, Medical University Vienna, Vienna, Austria.

Table of contents:

Definitions & Indications for Eculizumab therapy 2

Supplemental Table 1 3

Supplemental Table 2 4

References 5

**Definitions**

Anemia was defined as a hemoglobin level < 12.5 g/dL for women and < 13.5 g/dL for men. Mechanical hemolysis was defined by the presence of schistocytes in the peripheral blood smear. Furthermore, a decrease of haptoglobin levels and elevation of lactate dehydrogenase levels were considered. A direct Coombs-Test was performed in all patients. Thrombocytopenia was defined as a platelet count < 150 G/L. If thrombocytopenia was not present, a decline of thrombocyte counts of >25% within 48 hours was also counted as a sign of TMA. Renal response to eculizumab therapy was defined as decrease in serum creatinine concentrations.

CKD stages were graded according to the current Kidney Disease Improving Global Outcomes (KDIGO) guidelines using the serum creatinine based Chronic Kidney Disease Epidemiology Collaboration (CKD-EPI) equation to calculate estimated glomerular filtration rates.[1,2]

Non-response to PE or PI were defined as a lack of improvement in kidney function as measured by serum creatinine levels and calculated as eGFR.

**Indications for eculizumab therapy**

The most frequent indication for terminal complement blockade was cTMA (n=19, 40%) and paroxysmal nocturnal hemoglobinuria (n=15, 32%). Other indications were sTMA (n=6, 13%) and C3G (n=3, 6%) followed by systemic lupus erythematodes, myasthenia gravis, Shiga-toxin associated HUS and hemolysis of unclear origin after bone-marrow transplantation (each n=1, 2%)

**Supplemental Table 1.** Development of CKD stages in patients.

Characteristic Baseline 4 weeks 6 months Last FU

All patients

CKD stage 1 0 2 (8.7%) 0 3 (13%)

2 3 (13%) 3 (13%) 4 (17.4%) 1 (4.3%)

3a 1 (4.3%) 1 (4.3%) 4 (17.4%) 2 (8.7%)

3b 0 1 (4.3%) 3 (13%) 7 (30.4%)

4 7 (30.4%) 5 (21.7%) 6 (26.1%) 4 (17.4%)

5 3 (13%) 4 (17.4%) 0 2 (8.7%)

5d 9 (39.1%) 6 (26.1%) 6 (26.1%) 2 (8.7%)

cTMA

CKD stage 1 0 2 (13.3%) 0 3 (20%)

2 2 (13.3%) 2 (13.3%) 3 (20%) 1 (6.7%)

3a 1 (6.7%) 1 (6.7%) 4 (26.7%) 2 (13.3%)

3b 0 1 (6.7%) 2 (13.3%) 3 (20%)

4 4 (26.7%) 3 (20%) 3 (20%) 3 (20%)

5 2 (13.3%) 3 (20%) 0 1 (6.7%)

5d 6 (40%) 3 (20%) 2 (13.3%) 1 (6.7%)

sTMA

CKD stage 1 0 0 0 0

2 0 0 0 0

3a 0 0 0 0

3b 0 0 0 0

4 2 (33%) 2 (33%) 1 (16.7%) 4 (66.7%)

5 1 (16.7%) 1 (16.7%) 3 (50%) 1 (16.7%)

5d 3 (50%) 3 (50%) 2 (33%) 1 (16.7%)

Abbreviations: CKD, chronic kidney disease; FU, follow-up; cTMA, complement gene-variant mediated thrombotic microangiopathy; sTMA, secondary thrombotic microangiopathy.

**Supplemental Table 2.** Adverse events while on eculizumab therapy.

Characteristic All patients cTMA sTMA C3G

Patient number 23 15 6 2

Adverse events, n 6 (26%) 2 (13.3%) 2 (33.3%) 0

Liver injury 1 (4.3%) 1 (6.7%) 0 0

Exanthema 1 (4.3%) 0 1 (16.7%) 0

Leukopenia 1 (4.3%) 0 1 (16.7%) 0

Worsening of arterial hypertension 1 (4.3%) 1 (6.7%) 0 0

Death 2 (8.6%) 1 (6.7%) 0 1 (50%)

Abbreviations: cTMA; complement-variant mediated thrombotic microangiopathy; sTMA, secondary thrombotic microangiopathy; C3G, C3-glomerulopathy.

References:

1. Stevens PE, Levin A, Kidney Disease: Improving Global Outcomes Chronic Kidney Disease Guideline Development Work Group M (2013) Evaluation and management of chronic kidney disease: synopsis of the kidney disease: improving global outcomes 2012 clinical practice guideline. Ann Intern Med 158 (11):825-830. doi:10.7326/0003-4819-158-11-201306040-00007

2. Levey AS, Stevens LA, Schmid CH, Zhang YL, Castro AF, 3rd, Feldman HI, Kusek JW, Eggers P, Van Lente F, Greene T, Coresh J, Ckd EPI (2009) A new equation to estimate glomerular filtration rate. Ann Intern Med 150 (9):604-612. doi:10.7326/0003-4819-150-9-200905050-00006
